# Longitudinal and reciprocal associations between financial strain, home characteristics and mobility in the National Health and Aging Trends Study

**DOI:** 10.1186/s12877-019-1340-7

**Published:** 2019-12-02

**Authors:** L. J. Samuel, S. L. Szanton, C. L. Seplaki, T. K. M. Cudjoe, R. J. Thorpe, E. M. Agree

**Affiliations:** 1 Johns Hopkins University School of Nursing, 525 North Wolfe St., Rm 426, Baltimore, MD 21205 USA; 2 Department of Health Policy and Management, Johns Hopkins University School of Nursing and Bloomberg School of Public Health, Baltimore, USA; 3 Department of Public Health Sciences, University of Rochester School of Medicine and Dentistry, Rochester, USA; 4 Department of Medicine, Johns Hopkins Center on Aging and Health, Division of Geriatric Medicine and Gerontology, Baltimore, USA; 5 Hopkins Center for Health Disparities Solutions, Bloomberg School of Public Health, and Department of Health, Behavior and Society, Bloomberg School of Public Health, Johns Hopkins University, Baltimore, USA; 6 Department of Sociology and Johns Hopkins Bloomberg School of Public Health, Department of Population, Family and Reproductive Health, Johns Hopkins University Krieger School of Arts and Sciences, Baltimore, USA

**Keywords:** Financial strain, Socioeconomic factors, Housing, Walking speed, Mobility limitation

## Abstract

**Background:**

Older adults need homes that suit their physical capacity. Financial strain may limit home repairs and modifications and prompt relocations; repairing, relocating or modifying may increase financial strain. Likewise, reciprocal relationships may exist between financial strain and home characteristics and mobility; financial strain and home characteristics may influence mobility and mobility declines may increase financial strain, limit home repairs and modifications and prompt relocations. We test cross-lagged associations between financial strain, home disorder, relocation, home modifications and mobility.

**Methods:**

In the National Health and Aging Trends Study, ability to complete a walking test, speed among those able to complete, financial strain, home disorder, relocating and modifying the home were recorded annually for 3 years (2012–2014). Structural equation models separately examined ability to walk and walking speed among those able, accounting for sociodemographic characteristics, social support, health prior health characteristics and autoregressive effects. Sampling weights accounted for the complex survey design and non-response over time.

**Results:**

In both models (*n* = 3234 and *n* = 2467), financial strain predicted greater home disorder and vice versa, but cross-lagged associations were not found with relocating and modifications. Greater home disorder predicted lower odds of ability to walk and slower speed among those able. Financial strain and home modifications predicted lower odds of ability to walk. Also, faster walking speed predicted lower odds of subsequent financial strain and lower subsequent home disorder scores and ability to walk predicted less subsequent home disorder and lower odds of relocating.

**Conclusions:**

Home disorder links financial strain with reduced mobility in a national sample of U.S. older adults. Cross-lagged associations between financial strain and home disorder and between home disorder and mobility suggest reciprocal effects that may accumulate over time. Also, financial strain, reduced mobility, relocations and modifications predicted greater home disorder. Together, these results highlight home disorder as a social determinant of mobility for older adults. Greater attention should be given to repairing and modifying home environments and supporting stable housing for older adults with financial strain.

## Background

Approximately one-third of U.S. older adults report financial strain [1]. Financial strain, defined as insufficient income to meet basic needs [2], is an indicator of socioeconomic disadvantage representing a lack of access to the resources needed to avoid or ameliorate the consequences of chronic illness and disability. Financial strain is associated with poorer physical function [3–6] and slower average walking speed [7] in older adults, but the mechanisms contributing to these associations are unclear. This is important because slower walking speed predicts earlier mortality, increased risk of disability and nursing home admission among older adults [8–10].

Older adults with financial strain may not be able to afford high-quality, stable housing and this may influence mobility. Low educational achievement is associated with greater home disorder, such as broken furniture and flooring, and home disorder is associated cross-sectionally with worse lower extremity function [11]. Lower income older adults move more often than their peers and relocating predicts incident functional limitations [12]. Despite describing home environmental conditions as key social determinants of walking mobility in older adults [13], there is a paucity of evidence examining housing [14, 15].

Older adults with financial strain may also be unable to modify their home environment to suit their physical capacity, which may limit mobility. Older adults living in less accessible homes report, on average, less independence [16] and less use of assistive devices [17], which may limit mobility. More specifically, barriers at the home entrance predict mobility limitations [18] in older adults. Home modifications, such as grab bars and ramps, may be cost prohibitive for individuals with limited economic resources; older adults with financial strain are less likely to have home modifications regardless of their functional status in cross-sectional studies [19]. Although cross-sectional studies show that older adults with disabilities are more likely to have home modifications than those without [20, 21], a longitudinal study showed that older adults living in homes with at least one modification at baseline were less likely to experience functional decline over 2 years than those lacking modifications [22]. Together, these results suggest that older adults may install home modifications if they anticipate or experience functional declines and that home modifications may help prevent further decline towards disability. Therefore, older adults with financial strain may lack housing that facilitates regular activity and mobility and this may accelerate functional decline, but this idea has not been tested.

Finally, reciprocal effects may exist whereby older adults with greater home disorder and those who either moved or modified their home are placed at greater risk for financial strain. Houses with greater disorder require more urgent repairs than higher quality housing. Therefore, older adults living in disordered housing may have greater risk for financial strain over time because of rising household expenses. Older adults experiencing mobility decline may be forced to change their home, regardless of their financial situation. They may either modify their home environment or move to a new home to maintain independence. Either change can be costly. This could increase financial risk for older adults living on a limited or fixed income. Therefore, home disorder, modifying the home or relocating could reciprocally increase risk of financial strain. Since financial strain may reduce mobility through numerous pathways (such as increasing the risk of chronic conditions and reducing medication adherence), these reciprocal effects could accelerate mobility decline over time. Together, these pathways through home environmental characteristics may contribute to the accumulated socioeconomic effects on mobility that have been found in other studies [4, 7]. However, there is a gap in testing reciprocal associations between financial strain and the home environment over time as they relate to mobility.

**Fig. 1:**
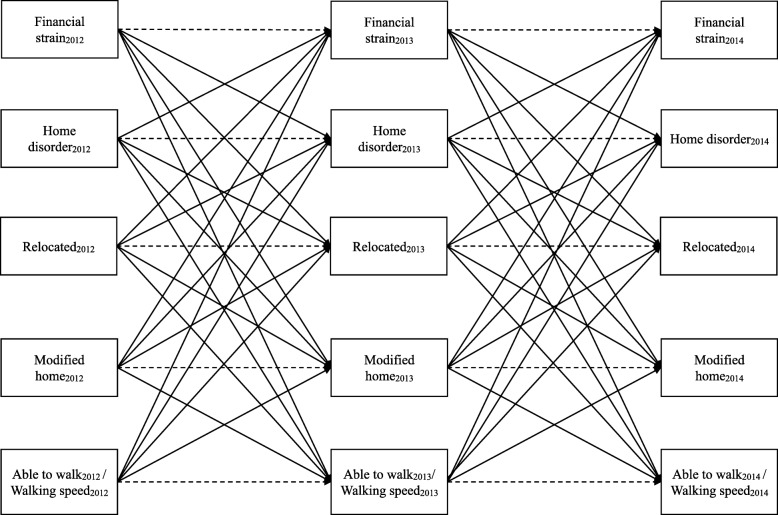
Structural equation model testing cross-lagged associations between financial strain, home disorder, relocating, modifying the home and mobility (i.e. ability to walk and speed among those able) over 3 years in the National Health and Aging Trends Study. Solid lines indicate hypothesized reciprocal inter-relationships over time between financial strain, home disorder, relocating, modifying the home and walking ability, which were constrained to be equal across years in the structural equation model. Dashed lines indicate autoregressive associations for each variable, which were not constrained to be equal across years. Correlations between contemporaneously measured financial strain, home disorder, relocating, modifying the home and walking are not shown but were estimated in the models

There is a need to understand the inter-relationships between financial strain, home environmental characteristics and mobility in community-dwelling older adults. Research in this area has timely policy implications because of new structures and incentives for payers and health systems to address modifiable social determinants of health, such as home environmental conditions [23]. This study tests hypothesized lagged reciprocal associations over time between financial strain, home disorder, relocating, modifying the home and mobility in 3 years of annual interview data taken from a nationally representative sample of community-dwelling Medicare beneficiaries aged 65 and older, as shown in Fig. 1.

## Methods

### Sample

The National Health and Aging Trends Study (NHATS) is a panel study of U.S. Medicare beneficiaries who were age 65 and older in 2011 [24]. In-home interviews are conducted annually by trained interviewers. Since financial strain was added in 2012, and since cross-lagged models are intended to model an underlying process that is stationary over time [25], these analyses are restricted to participants who remained community-dwelling through the 2014 interview. Of the 7197 community-dwelling NHATS participants who completed the 2011 interview, 5640, 4508 and 3692 participants completed interviews in 2012, 2013 and 2014, respectively; 942 participants died and another 2590 dropped out by 2014. Participants who either died or dropped out were older at baseline (mean age 80 vs. 74 years) and were more likely to have less than a high school level of education (26% vs. 15%) than those who remained in the study but did not differ by sex (60% vs. 56% female), race (10% vs. 8% black, 6% vs. 7% Hispanic and 2% vs. 4% other races) or mean income to poverty ratio (482% vs 272%). Differential baseline nonresponse and differential attrition between 2012 and 2014 were addressed using 2014 sampling weights, allowing inferences to be drawn to the 2014 population of U.S. Medicare beneficiaries aged 68 years and older.

### Measures

#### Walking ability

Values for mobility, financial strain, home disorder, relocating and modifying the home were recorded each year in NHATS. Mobility, the main outcome, was measured by a three-meter usual walking speed test with good reliability [9, 26]. Since older adults with limited mobility may be more sensitive to their home environment than those without limitations [27], we created two mobility variables. First, a binary variable compared participants who were able to complete the walking test to those who were unable or ineligible. Participants were ineligible for the walking test if they always used a wheelchair or scooter to get around inside home or if they reported they were unable to walk a short distance. Second, walking speed (meters/second) was recorded among participants who were able to complete the test. Participants were allowed to use an assistive device. The average of two test trials was used in analyses.

#### Financial strain and home environmental characteristics

Key exposures for these analyses include financial strain, relocating, modifying the home and home disorder. Participants were classified as having financial strain if they reported lacking money to pay the rent/mortgage, utility bills or medical/prescription bills or skipping meals because there was not enough money to buy food [28]. A dummy variable identified participants who modified their home. Self-reported home modifications included installing a ramp, elevator, stair lift, shower grab bar, bath seat, raised toilet seat or toilet grab bar in the past year. Older adults who relocated were identified by an indicator of whether the participant’s home address changed between annual interviews. A home disorder index summed up to seven indicators for peeling paint, evidence of pests, broken furniture, flooring in disrepair, tripping hazards, household clutter in the interview room and clutter elsewhere, as noted by the interviewer [11]. There were no missing data for home disorder index items. Prior to analyses, we truncated at four the more extreme values due to small counts. This avoids unstable estimates in structural equation models. Cronbach α ranged from 0.74–0.76 across years.

#### Covariates

Additional data used for these analyses include baseline (i.e. 2011) values for age (centered at 65 years), sex, race/ethnicity (analyzed as Black vs. all others and Hispanic vs. all others), educational achievement, and household income to poverty ratio and time variant values for height, BMI, chronic conditions and social support. The higher level of household educational achievement for the participant or a spouse/partner was used in these analyses, as in prior work [11] (analyzed as < high school, high school, some college, and ≥ Bachelor’s degree). Since financial strain is often correlated with income, but is intended to capture the sufficiency of income for daily needs and not just the absolute income level, this study accounted for income. In a process described elsewhere [29], income data was imputed by NHATS for 31% of participants with missing values and another 13% of participants who provided bracketed income ranges. We then further adjusted the income measure for baseline household size by dividing it by the relevant 2011 US Census Bureau poverty threshold for individuals over age 65 (e.g., the thresholds started at $10,788 annual income for a one-person household). We used this income to poverty threshold ratio in our analyses. Since changes in social isolation may affect both key study exposures and mobility in older adults, an indicator classified participants as having social isolation in 2012 and 2013 if they reported having no one to talk with about “important things”. For these analyses, previous health characteristics were also treated as confounders because they may worsen both key exposures and walking speed. However, since health characteristics over the study interval may mediate hypothesized relationships between exposures and mobility, we used height, BMI, and chronic condition values obtained in 2011 and 2012. Body mass index (BMI) was measured with self-reported height and weight. A medical condition summary score was created, summing the total number of medical conditions, as in prior work [11].

### Statistical analysis

Structural equation models were used to test hypothesized associations across the three study years. Structural models are ideal to simultaneously regress multiple dependent variables on multiple independent variables and account for reciprocal effects over time. Prior to building final structural models, bivariate associations were tested. Bivariate associations were assumed to be stationary across time (i.e. have constant effect over time) and were constrained to be equal across study years [25].

Two final structural equation models were built, one for ability to walk and another for walking speed to simultaneously test hypothesized cross-lagged associations (i.e. reciprocal associations over time). As shown in Fig. 1, each model tested associations for five separate dependent variables (financial strain, home disorder, relocating, modifying the home and mobility) that were each measured at two time points. Cross-lagged associations (i.e. reciprocal associations over time) between financial strain, home disorder, relocating, modifying the home and mobility were tested by regressing each variable on the prior year’s values for each of the other four variables. All associations adjusted for baseline covariates, including age, age^2^, sex, race/ethnicity, education, income to poverty ratio, social support and autoregressive effects, meaning that each dependent variable was regressed on the prior year’s value. Associations modeling financial strain, home disorder, relocations or modifications as the dependent variable additionally adjusted for one-year lagged social support values. Associations modeling mobility as the dependent variable additionally adjusted for two-year lagged values for height, chronic conditions and BMI. Based on standards for cross-lagged structural models, all pairwise cross-lagged associations were assumed to be stationary, or constant, over time. To impose the assumption of stationarity, each pairwise association was constrained to be equal across study rounds [25]. Constraints did not worsen model fit, based on chi-square difference tests for the walking test completion model (*p* = 0.09) and the walking speed model (*p* = 0.30).

Correlations between contemporaneously measured financial strain, home disorder, relocating, modifying the home and walking ability were estimated in the models [25]. Binary and ordinal dependent variables, including financial strain, home disorder, relocating, modifying the home and ability to walk were modeled using weighted least-squares with mean and variance adjustment, which is a robust estimator for categorical data. All analyses were conducted in Mplus version 8.3. Sample weights, accounting for sampling design, baseline nonresponse and attrition were used such that the results were considered to be representative of the population of Medicare beneficiaries over 68 years in 2014. Model fit was evaluated based on the root mean square error of approximation (RMSEA); values < 0.05 are considered to have good fit [30].

## Results

Approximately 5% of the weighted sample reported financial strain in 2012. Older adults with financial strain in 2012 had lower average incomes (172% poverty level vs. 507% poverty) and were more likely to be female (65% vs. 55%), black (20% vs. 7%) or Hispanic (20% vs. 6%), and other races (6% vs. 4%), have less than a high school level of education (33% vs. 13%) and greater home disorder (mean score 1.25 vs. 0.50) than their peers (Table 1). However, they did not differ by age or rates of relocating or modifying the home. Older adults reporting financial strain in 2012 had a similar rate of ability to walk (92% vs. 94%) but those who were able to walk had slower average speed (0.66 m/s vs. 0.82 m/s) that year. Bivariate associations between financial strain, home disorder, relocating, modifying the home and walking outcomes, constrained across study rounds, are shown in Additional file 1: Table S1. Final structural equation models for ability to walk and walking speed demonstrated good model fit (RMSEA = 0.023 and 0.027, respectively).

**Table 1:** Selected 2012 characteristics of National Health and Aging Trends Study community–dwelling participants who were followed until 2014, by 2012 financial strain

		No financial strain (*n* = 3221)	Financial strain (*n* = 212)	*p* value
Mean baseline age		74	73	0.321
Mean income: poverty		5.13	1.64	< 0.001
Educational achievement %	< high school	13	33	< 0.001
	high school	21	24	
	some college	31	31	
	≥ Bachelor’s	35	12	
Gender %	Male	45	35	0.015
	Female	55	65	
Race/ethnicity %	White	83	54	(ref.)
	Black	7	20	< 0.001
	Hispanic	6	20	< 0.001
	Other	4	6	0.041
Relocated %	No	97	94	0.094
	Yes	3	6	
Modified home %	No	86	82	0.282
	Yes	14	18	
Mean home disorder index		0.50	1.25	< 0.001
Able to walk %	No	6	8	0.184
	Yes	94	92	
Mean speed among those able (m/s) (*n* = 3034)		0.82	0.66	< 0.001

Note: 2014 sampling weights were used to represent the population of Medicare beneficiaries aged 68 years and older

In the final adjusted structural equation model including ability to walk, financial strain had cross-lagged associations with home disorder, but not with relocating or modifying the home as hypothesized (Table 2 and depicted in Additional file 2: Figure S1). Those with greater home disorder scores were more likely to experience financial strain in the subsequent year (B = 0.130, SE = 0.025) and older adults with financial strain had higher home disorder scores at the next annual interview (B = 0.197, SE = 0.043). Also, those who relocated (B = − 0.148, SE = 0.064) and those able to walk (B = − 0.128, SE = 0.059) had lower subsequent home disorder scores and those who modified their home (B = 0.090, SE = 0.033) had higher home disorder scores. Older adults with financial strain had higher odds (B = 0.182, SE = 0.049) and those able to walk had lower odds (B = − 0.172, SE = 0.044) of relocating in the subsequent year. Financial strain (B = − 0.145, SE = 0.057), greater home disorder (B = − 0.085, SE = 0.020) and home modifications (B = − 0.091, SE = 0.045) predicted reduced odds of ability to walk at the next year’s exam, as hypothesized but relocating did not.

**Table 2:** Cross-lagged and autoregressive associations between financial strain, home disorder, relocating and modifying home and ability to walk among community-dwelling National Health and Aging Trends Study participants followed from 2012 to 2014 (*n* = 3234)

	Financial strain_t_^a^ B (SE)	Home disorder_t_^a^ B (SE)	Relocated_t_^a^ B (SE)	Modified home_t_^a^ B (SE)	Able to walk_t_^b^ B (SE)
Independent variable					
Financial strain_t-1_	1.472 (0.099)** 0.824 (0.072)**	0.197 (0.043)**	0.182 (0.049)**	−0.014 (0.050)	−0.145 (0.057)*
Home disorder_t-1_	0.130 (0.025)**	0.678 (0.024)** 0.832 (0.038)**	0.005 (0.022)	0.039 (0.020)	−0.085 (0.020)**
Relocated_−1_	0.029 (0.074)	−0.148 (0.064)*	0.853 (0.136)** 0.380 (0.072)**	−0.006 (0.067)	−0.096 (0.074)
Modified home_t-1_	0.001 (0.067)	0.090 (0.033)**	0.015 (0.043)	0.713 (0.089)** 0.401 (0.050)**	−0.091 (0.045)*
Able to walk_t-1_	−0.133 (0.068)	−0.128 (0.059)*	− 0.172 (0.044)**	0.013 (0.051)	1.312 (0.113)** 0.885 (0.102)**

Note: Adjusted for baseline age, age-squared, sex, black race, Hispanic ethnicity, education and income and one-year lagged values of financial strain, home disorder, relocating, modifying home and ability to walk in a structural equation model with good fit (RMSEA = 0.023). Unstandardized coefficients are presented. Cross-lagged were associations constrained across study years. 2014 sampling weights were used

^a^ Additionally adjusted for presence of social support

^b^ Additionally adjusted for two-year lagged values of height, chronic conditions and BMI

*p < 0.05

**p < 0.01

Since this study tested hypothesized pathways between financial strain and mobility, we also quantified the indirect effects of financial strain on subsequent mobility that are attributable to home disorder, relocating or modifying the home. In the final adjusted structural equation model, there was a statistically significant total effect of financial strain on ability to walk 2 years later (B = − 0.375, SE = 0.138, *p* = 0.006, data not shown), attributable, in part, to interim differences in home disorder (B = − 0.017, SE = 0.005, *p* = 0.001) but not interim relocations or home modifications.

In the final adjusted structural equation model including walking speed among those able to walk in 2014, financial strain had cross-lagged associations with home disorder, but not with relocating or modifying the home as hypothesized (Table 3 and depicted in Additional file 3: Figure S2). Older adults with greater home disorder scores were more likely to experience financial strain (B = 0.150, SE = 0.030) and those with financial strain had higher home disorder scores (B = 0.173, SE = 0.040) in the subsequent year. Also, relocating predicted less home disorder (B = − 0.165, SE = 0.069) and home modifications predicted higher home disorder scores (B = 0.102, SE = 0.037). Greater home disorder (B = − 0.008, SE = 0.002) predicted slower walking speed at the next annual interview as hypothesized but financial strain, relocating and modifying the home did not. Older adults with faster walking speed had lower odds of financial strain (B = − 0.639, SE = 0.171) and lower home disorder scores (B = − 0.300, SE = 0.118) in the subsequent year. Also, greater home disorder predicted higher odds of home modifications in the subsequent year (B = 0.047, SE = 0.024). There were no statistically significant total effect of financial strain on walking speed 2 years later.

**Table 3:** Cross-lagged and autoregressive associations between financial strain, home disorder, relocating and modifying home and walking speed (m/s) among community-dwelling National Health and Aging Trends Study participants followed from 2012 to 2014 and able to complete the walking test in 2014 (*n* = 2467)

	Financial strain_t_^a^ B (SE)	Home disorder_t_^a^ B (SE)	Relocated_t_^a^ B (SE)	Modified home_t_^a^ B (SE)	Walking speed (m/s)_t_^b^ B (SE)
Independent variable					
Financial strain_t-1_	1.600 (0.124)** 0.774 (0.095)**	0.173 (0.040)**	0.115 (0.059)	−0.040 (0.054)	0.002 (0.005)
Home disorder_t-1_	0.150 (0.030)**	0.704 (0.031)** 0.860 (0.052)**	−0.020 (0.031)	0.047 (0.024)*	−0.008 (0.002)**
Relocated_t-1_	0.004 (0.092)	−0.165 (0.069)*	1.009 (0.141)** 0.469 (0.098)**	−0.063 (0.070)	−0.007 (0.006)
Modified home_t-1_	−0.098 (0.073)	0.102 (0.037)**	−0.051 (0.051)	0.686 (0.097)** 0.419 (0.057)**	−0.006 (0.004)
Walking speed (m/s)_t-1_	−0.639 (0.171)**	−0.300 (0.118)*	− 0.139 (0.170)	−0.234 (0.147)	0.671 (0.020)** 0.829 (0.023)**

Note: Adjusted for baseline age, age-squared, sex, black race, Hispanic ethnicity, education and income and one-year lagged values of financial strain, home disorder, relocating, modifying home and walking speed in a structural equation model with good fit (RMSEA = 0.027). Unstandardized coefficients are presented. Cross-lagged associations were constrained across study years. 2014 sampling weights were used

^a^ Additionally adjusted for presence of social support

^b^ Additionally adjusted for two-year lagged values of height, chronic conditions and BMI

*p < 0.05

**p < 0.01

## Discussion

These results identify potential pathways linking financial strain and home disorder with reduced mobility in a nationally representative sample of older adults. We found partial support for both study hypotheses. First, we found reciprocal associations over time between financial strain with home disorder, but not between financial strain with either relocating or modifying the home. Second, home disorder predicted both reduced ability and slower walking speed among those able to walk, and financial strain and home modifications predicted reduced ability to walk but relocations did not predict subsequent mobility in either model. Importantly, this study also found that less mobility predicted higher odds of financial strain, relocating and modifications and greater home disorder, suggesting feedback loops. Finally, relocating predicted less subsequent home disorder whereas home modifications predicted more home disorder. Together, these results show that home disorder is linked with financial strain, relocations, home modifications, and walking ability suggesting that it is an important social determinant of mobility declines among community-dwelling adults. Also, feedback loops between mobility with financial strain and home environmental characteristics suggest that interventions addressing financial strain and home environmental conditions may prevent a downward spiral of decline among vulnerable older adults.

There are potential reasons for the results related to the first hypotheses. Greater home disorder may increase the risk of financial strain because of relatively higher home maintenance costs over time. Alternatively, home disorder may be associated with subsequent financial strain if they share a common cause, such as a lack of financial, social and health-related resources that can help an older adult to maintain both their home environment and their financial security. Either way, these results suggest a reciprocal effect between financial strain and home disorder that may accumulate over time. The feedback loop is noteworthy because financial strain is associated with numerous other adverse health outcomes such as greater risk for obesity, disability and malnutrition, poorer self-rated health, increased depressive symptoms, and earlier mortality [5, 6, 31–34]. Also, home disorder partially accounted for indirect associations between financial strain at baseline and ability to walk 2 years later. These results highlight the importance of home disorder in relation to financial disparities in mobility among older adults.

In addition to the hypothesized relationships between financial strain and home disorder, this study also found that relocations and home modifications predicted subsequent home disorder. These results suggest that home disorder is closely inter-related with other social determinants of health. However, we also found different results related to relocating versus modifying the home; modifying the home predicted greater subsequent home disorder, whereas relocating predicted less. This matters because older adults with mobility declines may face decisions about whether to modify their existing home or relocate to a new home. Our results suggest that greater attention should be paid to assist older adults aging at home to modify their home in ways that do not increase disorder. Based on reciprocal relationships between mobility and home disorder in this study, such interventions may ameliorate a progressive cycle of mobility decline among vulnerable older adults. These results also suggest that older adults who experience mobility declines may benefit from assistance to maintain their home and to relocate and/or modify their home to facilitate aging in place.

There are also potential reasons for results showing that greater home disorder, financial strain and home modifications predict mobility. Home disorder may limit indoor regular physical activity which could speed incident disability [35]. Alternatively, associations between home disorder with subsequent mobility may be due to underlying changes in health status and physical capacity in older adults. Together with other results, our results suggest that modifications cannot improve an individual’s mobility, but they may minimize functional decline [22] by enabling an older adult with declining ability to maintain independence in their home.

These results provide empirical support for fundamental cause theory [36]. Fundamental cause theory posits that those with greater access to resources will always experience better health even though the common diseases and intervening mechanisms may change over time and across settings [36, 37]. In this study, home disorder was inter-related with other social determinants of health and feedback loops were found that may exacerbate health declines among vulnerable older adults. As examples, older adults with declining mobility may relocate, modify and/or may be less able to maintain their home and may experience greater risk of financial strain over time. Feedback loops may be due to decreased physical capacity to maintain the home and/or greater competing needs related to health decline. Since home disorder represents a modifiable pathway, these results have important implications for interventions and policy. Reciprocal associations between financial strain and home disorder over time suggest that effects on mobility may accumulate over time. Therefore, it is likely never too late to either prevent or minimize mobility declines by targeting home disorder in interventions for financially strained older adults.

These results build on prior studies in two ways. First, these results identify home disorder, home modification and relocation as potential targets for home environmental interventions. Prior work has shown that home environments are modifiable. Home renovations and modifications, such as making repairs, installing ramps, handrails and grab bars can improve an older adult’s ability to function independently in their home [38, 39]. Although several home based interventions have installed modifications such as grab bars, railings and ramps [38, 40, 41], only one made home repairs [42], which could improve home disorder, and none addressed relocating. This study and other work [11] identify home disorder as a potential mechanism contributing to socioeconomic disparities in physical function, highlighting the need for additional interventions to repair the home and remove clutter for low income older adults. Together, these studies highlight the need for multi-component home interventions focused on reducing home disorder and ensuring stable housing for low income older adults.

Secondly, since renovations and modifications can be costly, these results show that older adults with financial strain should be prioritized for such interventions. These results highlight the importance of improving access to stable, affordable and high quality housing for financially strained older adults, especially those experiencing mobility limitations. Policies and programs can be developed and strengthened to provide financial support for older adults in need of home repairs and modifications. With the U.S. health care system shifting from paying for the number and types of visits and procedures, to paying for better population health outcomes, health providers may start to focus on the fixable social determinants that have proven return on investment [23, 43, 44]. Results from this study and others may help reorient payers towards home modification and repair.

### Limitations

Study results may be affected by survival bias in this sample of older adults. Financial strain and home environmental characteristics were measured with few indicators, which could induce measurement error and binary indicators do not quantify financial strain, home modifications or relocations. This study did not measure social or built environmental characteristics that may influence mobility. This study is strengthened by using walking speed test results as outcomes, which are objective measures of mobility. Also, selection bias was addressed, in part, by the including participants who were unable to complete the walking test in analyses.

## Conclusions

In conclusion, this study found reciprocal effects of financial strain and home disorder as they relate to mobility in older adults. Also, financial strain, reduced mobility, relocations and modifications predicted greater home disorder, suggesting that home disorder is closely inter-related with other social determinants of health. Finally, mobility is reciprocally associated with financial strain, home disorder, relocations and home modifications over time, suggesting that interventions may prevent a downward spiral of decline.. Since home environmental conditions are modifiable, these results have important implications for policies and public health interventions. Interventions to assist financially strained older adults with home modifications and efforts to improve access to high quality affordable housing may minimize mobility decline for vulnerable older adults.

## Supplementary information

**Additional file 1: Table S1.** Bivariate one-year lagged associations between financial strain, home disorder, relocating, modifying home and mobility among National Health and Aging Trends Study participants (2012–2014).

**Additional file 2: Figure S1.** Structural equation model testing cross-lagged associations between financial strain, home disorder, relocating, modifying the home and ability to walk over three years in the National Health and Aging Trends Study, 2012-2014 (n= 3234). Solid lines indicate statistically significant associations; dashed lines indicate that there was no statistically significant association. Cross-lagged were associations constrained across study years and concurrent measures were allowed to be correlated (not shown in model). 2014 sampling weights were used. All associations adjusted for baseline age, age-squared, sex, black race, Hispanic ethnicity, education and income. Associations with financial strain, home disorder, relocation, and home modifications as dependent variables were additionally adjusted for presence of social support. Associations with ability to walk as dependent variable were additionally adjusted for two-year lagged values of height, chronic conditions and BMI.

**Additional file 3: Figure S2.** Structural equation model testing cross-lagged associations between financial strain, home disorder, relocating, modifying the home ability to walk over three years in the National Health and Aging Trends Study, 2012-2014 (n= 2467). Solid lines indicate statistically significant associations; dashed lines indicate that there was no statistically significant association. Cross-lagged were associations constrained across study years and concurrent measures were allowed to be correlated (not shown in model). 2014 sampling weights were used. All associations adjusted for baseline age, age-squared, sex, black race, Hispanic ethnicity, education and income. Associations with financial strain, home disorder, relocation, and home modifications as dependent variables were additionally adjusted for presence of social support. Associations with ability to walk as dependent variable were additionally adjusted for two-year lagged values of height, chronic conditions and BMI.

## Data Availability

The data supporting the conclusions of this article are available at https://www.nhats.org/.

## Abbreviations

BMI: Body mass index
NHATS: National Health and Aging Trends Study
U.S.: United States
