# Supplementary material for: Longitudinal and reciprocal associations between financial strain, home characteristics and mobility in the National Health and Aging Trends Study
Source: BMC Geriatr. 2019 Dec 2;19:338. doi: 10.1186/s12877-019-1340-7 (PMC6888936; doi:10.1186/s12877-019-1340-7)
Supplement: Supplementary file 1 — Additional file 1: Table S1. Bivariate one-year lagged associations between financial strain, home disorder, relocating, modifying home and mobility among National Health and Aging Trends Study participants (2012–2014). [file 12877_2019_1340_MOESM1_ESM.docx]

Supplemental Table 1

Bivariate one-year lagged associations between financial strain, home disorder, relocating, modifying home and mobility among National Health and Aging Trends Study participants (2012-2014)

|  | Financial strain_t_  B (SE) | Home disorder_t_  B (SE) | Relocated_t_  B (SE) | Modified home_t_  B (SE) | Able to walk_t_  B (SE) | Walking speed_t_ (m/s) B (SE) |
| --- | --- | --- | --- | --- | --- | --- |
| **Independent variable** |  |  |  |  |  |  |
| Financial strain_t-1_ | N/A | 0.539 (0.069)** | 0.126 (0.094) | 0.066 (0.073) | -0.180 (0.075)* | -0.053 (0.013)* |
| Home disorder_t-1_ | 0.128 (0.023)** | N/A | 0.042 (0.031) | 0.026 (0.020) | -0.121 (0.016)** | -0.019 (0.003)** |
| Relocated_t-1_ | 0.223 (0.109)* | -0.057 (0.067) | N/A | 0.010 (0.088) | -0.531 (0.083)** | -0.038 (0.013)** |
| Modified home_t-1_ | 0.152 (0.067)* | 0.196 (0.036)** | 0.114 (0.065) | N/A | -0.268 (0.049)** | -0.023 (0.007)** |
| Able to walk_t-1_ | -0.035 (0.012)** | -0.285 (0.061)** | -0.140 (0.065)* | -0.075 (0.074) | N/A | N/A |
| Walking speed_t-1_ (m/s) | -0.089 (0.013)** | -0.339 (0.090)** | -0.283 (0.192) | -0.393 (0.135)** | N/A | N/A |

Note: Unstandardized coefficients are presented. Lagged effects are constrained across study years. 2014 sampling weights were used to represent the population of Medicare beneficiaries aged 68 years and older. N's ranged from 3794 to 4414, except for associations with walking speed, which was only measured among those able to complete the walking test (N’s ranges from 3176 to 3794).

*p<0.05

**p<0.01
