# Supplementary material for: Longitudinal and reciprocal associations between financial strain, home characteristics and mobility in the National Health and Aging Trends Study
Source: BMC Geriatr. 2019 Dec 2;19:338. doi: 10.1186/s12877-019-1340-7 (PMC6888936; doi:10.1186/s12877-019-1340-7)
Supplement: Supplementary file 3 — Additional file 3: Figure S2. Structural equation model testing cross-lagged associations between financial strain, home disorder, relocating, modifying the home ability to walk over three years in the National Health and Aging Trends Study, 2012-2014 (n= 2467). Solid lines indicate statistically significant associations; dashed lines indicate that there was no statistically significant association. Cross-lagged were associations constrained across study years and concurrent measures were allowed to be correlated (not shown in model). 2014 sampling weights were used. All associations adjusted for baseline age, age-squared, sex, black race, Hispanic ethnicity, education and income. Associations with financial strain, home disorder, relocation, and home modifications as dependent variables were additionally adjusted for presence of social support. Associations with ability to walk as dependent variable were additionally adjusted for two-year lagged values of height, chronic conditions and BMI. [file 12877_2019_1340_MOESM3_ESM.docx]

Financial strain_t_

Walking speed_t_

Modified home_t_

Home disorder_t_

Home disorder _t-1_

Modified home_t-1_

Walking speed_t-1_

Financial strain_t-1_

Relocated_t_

Relocated _t-1_

Supplementary Figure 2

Structural equation model testing cross-lagged associations between financial strain, home disorder, relocating, modifying the home ability to walk over three years in the National Health and Aging Trends Study, 2012-2014 (n= 2467). Solid lines indicate statistically significant associations; dashed lines indicate that there was no statistically significant association. Cross-lagged were associations constrained across study years and concurrent measures were allowed to be correlated (not shown in model). 2014 sampling weights were used. All associations adjusted for baseline age, age-squared, sex, black race, Hispanic ethnicity, education and income. Associations with financial strain, home disorder, relocation, and home modifications as dependent variables were additionally adjusted for presence of social support. Associations with ability to walk as dependent variable were additionally adjusted for two-year lagged values of height, chronic conditions and BMI.
